# Novel c-Met inhibitor suppresses the growth of c-Met-addicted gastric cancer cells

**DOI:** 10.1186/s12885-016-2058-y

**Published:** 2016-01-22

**Authors:** Chi Hoon Park, Sung Yun Cho, Jae Du Ha, Heejung Jung, Hyung Rae Kim, Chong Ock Lee, In-Young Jang, Chong Hak Chae, Heung Kyoung Lee, Sang Un Choi

**Affiliations:** Bio-Organic Science Division, Korea Research Institute of Chemical Technology, PO Box 107, Daejeon, 305-600 Republic of Korea; Medicinal Chemistry and Pharmacology, Korea University of Science and Technology, Daejeon, 305-350 Republic of Korea

**Keywords:** c-Met Inhibitor, KRC-00715, Gastric cancer, Oncogene addiction

## Abstract

**Background:**

c-Met signaling has been implicated in oncogenesis especially in cells with *c*-*met* gene amplification. Since 20 % of gastric cancer patients show high level of c-Met expression, c-Met has been identified as a good candidate for targeted therapy in gastric cancer. Herein, we report our newly synthesized c-Met inhibitor by showing its efficacy both in vitro and in vivo.

**Methods:**

Compounds with both triazolopyrazine and pyridoxazine scaffolds were synthesized and tested using HTRF c-Met kinase assay. We performed cytotoxic assay, cellular phosphorylation assay, and cell cycle assay to investigate the cellular inhibitory mechanism of our compounds. We also conducted mouse xenograft assay to see efficacy in vivo.

**Results:**

KRC-00509 and KRC-00715 were selected as excellent c-Met inhibitors through biochemical assay, and exhibited to be exclusively selective to c-Met by kinase panel assay. Cytotoxic assays using 18 gastric cancer cell lines showed our c-Met inhibitors suppressed specifically the growth of c-Met overexpressed cell lines, not that of c-Met low expressed cell lines, by inducing G1/S arrest. In *c*-*met* amplified cell lines, c-Met inhibitors reduced the downstream signals including Akt and Erk as well as c-Met activity. In vivo Hs746T xenograft assay showed KRC-00715 reduced the tumor size significantly.

**Conclusions:**

Our in vitro and in vivo data suggest KRC-00715 is a potent and highly selective c-Met inhibitor which may have therapeutic potential in gastric tumor with c-Met overexpression.

**Electronic supplementary material:**

The online version of this article (doi:10.1186/s12885-016-2058-y) contains supplementary material, which is available to authorized users.

## Background

Oncogene addiction, which was first proposed by Dr. Bernard Weinstein, offers a rationale for the recent targeted therapy in cancer biology [1]. Oncogene addiction refers to the phenomenon which the tumorigenesis is dependent on a specific cellular signal. In view of oncogene addiction, RTKs have been highlighted as promising therapeutic targets against cancer for their links with various malignancies [2]. The clinical success of tyrosine kinase inhibitors, such as imatinib and erlotinib, prompted intensive studies to identify additional oncogenic proteins for the targeted therapy. c-Met is one of the most genetically amplified and dysregulated RTKs in a subset of solid tumor implicating it as a prominent therapeutic target.

The c-MET receptor tyrosine kinase forms a heterodimer and consists of an extracellular α-chain and a membrane-spanning β-chain [3, 4]. c-MET is the only known high-affinity receptor for HGF [5]. Upon HGF binding, c-Met auto-phosphorylation happens on Y1234 and Y1235 within the activation loop of kinase domain, which promotes the kinase activity. Phosphorylated Y1349 and Y1356 serve as docking sites for the intracellular adapters which transmit signals downstream [6, 7]. Both c-Met and HGF are required for normal mammalian development [8]. After birth, c-Met activation by HGF is likely to be involved in epithelial-mesenchymal transition (EMT), as well as hepatic, renal and epidermis regeneration [9, 10]. c-Met overexpression by gene amplification has been reported in a number of solid tumors, and the resultant aberrant c-Met signaling appears to promote the growth, maintenance, survival and progression of cancer [11]. ShRNA-mediated c-Met knockdown induced significant growth inhibition in *c*-*met* amplified cell lines, whereas it had no effect on the cell lines without *c*-*met* amplification [12]. It strongly suggests the overexpression of c-Met by genomic amplification confers the constitutive activity on c-Met kinase, which eventually allows the cells to be exclusively dependent on c-Met signaling for proliferation and survival [12, 13]. It has been reported that 4 % of esophageal and 4 % of lung cancer patients have amplified *c*-*met* gene. Moreover, a large number of reports identified *c*-*met* amplification even in 10–20 % of gastric cancer [14–18]. It means c-Met is a most relevant target for gastric cancer therapy over other malignancies [19].

Gastric cancer is the second leading cause of cancer related mortality worldwide with the incidence of 18.9/100,000/year [20]. Molecules targeting EGFR, VEGF, PI3K/Akt/mTor signal pathway, and c-Met pathway have been investigated for molecular targeted therapy for gastric cancer [21]. Especially, c-Met has been fairly highlighted as a promising target in gastric cancer, for several papers described significant growth suppression by c-Met inhibitors [22–24].

Various approaches have been conducted to inhibit the aberrant c-Met kinase activity, such as c-Met biologics, HGF antagonist peptides, and HGF antibodies as well as small molecule inhibitors [25–29]. Here, we introduce novel potent small molecule inhibitor of c-Met and demonstrate the excellence of our compounds by showing in vitro and in vivo results.

## Methods

### Compounds and reagents

KRC-00509 and KRC-00715 were synthesized according to the procedures published in patent, KR2012-0022541. All compounds including crizotinib were dissolved in DMSO. Compounds were formulated in 20 % PEG-400, 3 % Tween-80, 77 % distilled water for all in vivo studies. Kinase domain of c-Met was purchased from CarnaBio Science (JAPAN).

### c-Met in vitro enzyme assay

Experiment procedure was followed by the manufactured instruction (Cisbio, France). The reaction was initiated by ATP addition to a mixture containing the c-Met enzyme, peptide substrates, and inhibitors. After 30 min, EDTA containing solution was added to stop the reaction. EDTA containing solution has Europium conjugated anti-phosphoresidue antibody and SA-XL665 for the detection of the phosphorylated peptide product. After 1 h incubation, fluorescence was measured with 337 nm excitation and dual 665 and 620 nm emission of the Envision reader. IC_50_ was calculated using GraphPad Prism version 5 for Windows. The curves were fit using a nonlinear regression model with a log (inhibitor) versus response formula.

### Cell culture

All cell lines used in this paper, except Hs746T, were purchased from Korean Cell Line Bank (KCLB, Korea). Hs746T cell line was purchased from ATCC. These are all gastric adenocarcinoma cells. SNU-5, SNU-620, SNU-638, MKN-45, and Hs746T cell lines show high expression of c-Met, whereas others show low level of c-Met. These cell lines were maintained in RPMI 1640 medium supplemented with 10 % FBS (HyClone, US) using a humidified incubator with 5 % CO2 at 37 °C.

### Antibodies and immunoblotting

The following antibodies were obtained from Cell Signaling Technology: c-Met (Catalog No. 3127), phospho c-Met tyrosine 1234/1235 (Catalog No. 3129), phospho-Erk threonine 202/204 (Catalog No. 4370), phospho-Akt serine 473 (Catalog No. 4060), phospho-tyrosine (Catalog No. 9416). Tubulin antibody (Catalog No. T6199) was purchased from Sigma-Aldrich. HRP-conjugated anti-mouse (Catalog No. NCI1430KR), and HRP-conjugated anti-rabbit (Catalog No. NCI1460KR) antibodies were obtained from Thermo Scientific. For immunoblotting, cells were washed in PBS, lysed in 1 X sample buffer (50 mmol/L Tris–HCl (pH 6.8), 10 % glycerol, 2 % SDS, 3 % β-mercaptoethanol), and boiled for 10 min. Lysates were subjected to SDS-PAGE followed by blotting with the indicated antibodies and detection by Western blotting substrate ECL reagent (Thermo Scientific). Images were quantified using a LAS3000 instrument and Image Lab software.

### Cell cytotoxicity assay

For viability experiments, cells were seeded in 96-well plates at 30 % confluency and exposed to chemicals the next day. After 72 h, WST-1 reagent was added and absorbance at 450 nm was measured on a Spectramax spectrophotometer (Molecular Devices, US) according to the manufacturer's instructions. IC_50_s were calculated using GraphPad Prism version 5 for Windows. The curves were fit using a nonlinear regression model with a log (inhibitor) versus response formula.

### Cell cycle analysis

We followed the manufacturer’s instruction to NucleoCounter NC-250 (chemometec, Denmark) to analyze the cell cycle distribution. Briefly, cells treated with vehicle or compounds for 24 h were suspended by lysis buffer supplemented with 10ug/ml DAPI. After 5 min incubation at 37 °C, cells were suspended by stabilization buffer. Cells were loaded into the chambers of the slide and were analyzed by NucleoCounter NC-250.

### Xenograft studies

Female athymic BALB/c (nu/nu) mice ( 6 weeks old) were obtained from Charles River of Japan. Animals were maintained under clean room conditions in sterile filter top cages and housed on high efficiency particulate air-filtered ventilated racks. Animals received sterile rodent chow and water ad libitum. All of the procedures were conducted in accordance with guidelines approved by the Laboratory Animal Care and Use Committee of Korea Research Institute of Chemical Technology. Hs746T cells (5 × 10^6^ in 100 μl) were implanted s.c. into the right flank region of each mouse and allowed to grow to the designated size. Once tumors reached an average volume of 200 mm^3^, mice were randomized and dosed via oral gavage daily with the indicated doses of compounds for 10 days. Mice were observed daily throughout the treatment period for signs of morbidity/mortality. Tumors were measured twice weekly using calipers, and volume was calculated using the formula: length × width^2^ × 0.5. Body weight was also assessed twice weekly. Significance differences between the treated versus the control groups (*P* ≤ 0.001) were determined using one-way ANOVA.

### Kinase panel assay

Kinase panel assay was done by Millipore. Kinases were incubated with peptide together with γ-^33^P-ATP (specific activity approx. 500 cpm/pmol, concentration as required). The reaction was initiated by the addition of the MgATP mix. After incubation for 40 min at room temperature, the reaction was stopped by the addition of 3 % phosphoric acid solution. 10 μl of the reaction was then spotted onto a P30 filtermat and washed three times for 5 min in 75 mM phosphoric acid and once in methanol prior to drying and scintillation counting.

## Results

### Identification of KRC-00509 and KRC-00715 as potent inhibitors against c-Met kinase

**Fig. 1:**
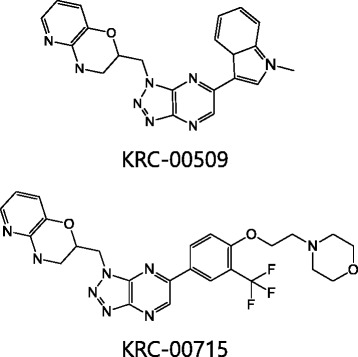
Chemical Structures of c-Met inhibitors

Cui et al. suggested that PF-04217903, which has triazolopyrazine scaffold, is effective for c-Met inhibition [30]. PF-04217903 binds to the hinge region through nitrogen of quinoline in a mono binding manner. We designed and synthesized hundreds of compounds having pyridoxazine instead of quinoline as a dual binder to hinge region. The enzymatic activities were determined by HTRF kinase assay using recombinant kinase domain of c-Met. Among the synthesized compounds, 2 chemicals in Fig. 1 showed most potent effect against c-Met. The IC_50_s of KRC-00509 and KRC-00715 were 6.3 nM and 9.0 nM, respectively (Table 1, Additional file 1: Figure S1). Crizotinib, which is under clinical trial for the c-Met over-expressed cancer patients, [31] had IC_50_ value of 2.2 nM. To know the cytotoxic activities of compounds on the c-Met-addicted gastric cancer cells, Hs746T cells were treated with these inhibitors for 3 days. Hs746T cell line has constitutively activated c-Met signaling by the amplified *c*-*met* gene and a splice site mutation of exon 14 [32]. KRC-00509 and KRC-00715 were highly cytotoxic to Hs746T cells with estimated cytotoxic IC_50_ values of approximately 3.4 nM and 39 nM, respectively. Biochemical and cellular cytotoxic data demonstrate that KRC-00509 and KRC-00715 are excellent c-Met inhibitors.

**Table 1:** IC_50_ of c-Met enzyme and cytotoxic assay

	IC_50_ (nM) in c-Met enzyme assay	IC_50_ (nM) in Hs746T cell cytotoxic assay
KRC-00509	6.3	3.4
KRC-00715	9.0	39
Crizotinib	2.2	10.0

### KRC-00715 is highly selective to c-Met over other tyrosine kinases

KRC-00715 was evaluated against 40 tyrosine kinases which represent each tyrosine kinase family in enzymatic assays by Millipore Inc. Surprisingly, as shown in Table 2, 1 μM KRC-00715 inhibited only c-Met. KRC-00715 never inhibited other tyrosine kinase. This data suggests that KRC-00715 is exclusively selective to c-Met.

**Table 2:** Kinase panel assay

	KRC-00715 @ 1 μM		KRC-00715 @ 1 μM		KRC-00715 @ 1 μM
Abl	104	Fer	100	Lyn	99
Ack1	96	Fes	97	Mer	105
ALK	101	FGFR1	107	Met	−2
Axl	107	Fgr	113	MuSK	106
Blk	102	Flt1	115	PDGFRα	121
Brk	96	Flt3	94	Ret	110
BTK	108	Fms	108	Ron	107
c-Ket	101	Fyn	106	Ros	115
CSK	116	Hck	81	Syk	110
DDR1	90	IGF-1R	92	Tie2	116
EGFR	111	IR	119	TrkA	99
EphA1	117	IRR	90	Yes	114
EphB1	104	JAK1	111		
FAK	105	KDR	110		

### c-Met inhibitors selectively suppress the growth of c-Met over-expressed gastric cancer cells

**Fig. 2:**
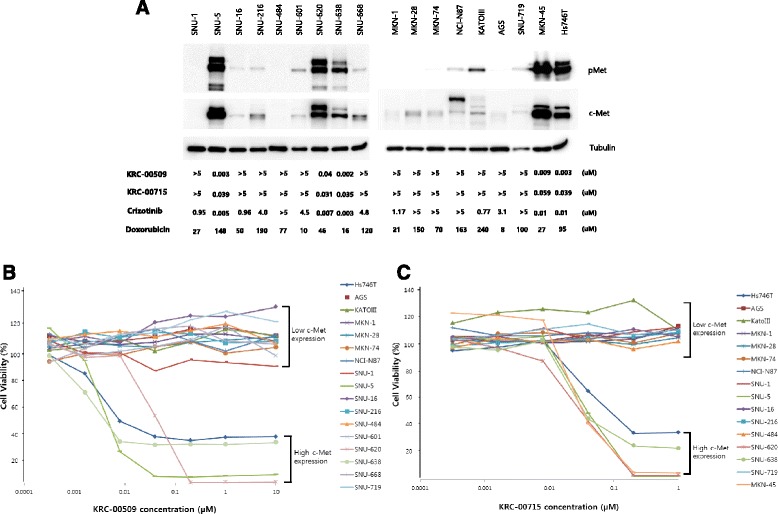
c-Met inhibitors are sensitive only to c-Met over-expressed cell lines. **a** The lysates of 18 gastric cancer cell lines were prepared for immunoblot with antibodies of c-Met and phospho c-Met (pY1234/1235). For cytotoxic IC_50_ measurement, cells were seeded on 96 well plates and were treated with chemicals for 72 h. After 72 h, WST-1 reagent was added to each well and absorbance at 450 nm was read with microplate reader (Molecular Device). IC_50_ was calculated with Prizm software program. The numbers indicate the cytotoxic micro molar IC_50_ (μM) of each compound. **b**-**c** The viability curves of each gastric cancer cell line treated with KRC-00509 (**b**) or KRC-00715 (**c**)

Using 18 gastric cancer cell lines, cytotoxic effects of our c-Met inhibitors were investigated. Figure 2a shows the c-Met expression levels in 18 gastric cancer cell lines. SNU-5, SNU-620, SNU-638, MKN-45, and Hs746T have high levels of total c-Met and phosphorylated c-Met proteins, whereas other 13 gastric cancer cell lines have extremely low levels of c-Met. 18 cancer cell lines were treated with doxorubicin, a well-known cytotoxic drug, for 72 h. There is no difference between c-Met over-expressed cell lines and c-Met low-expressed cell lines in doxorubicin-induced cytotoxic effect. In next step, these cell lines were treated with c-Met inhibitors for 72 h. Importantly, the cytotoxic IC_50_ values of c-Met inhibitors were less than or around 10 nM in c-Met over-expressed cell lines, whereas c-Met low-expressed cell lines were fully viable even at 5 μM of KRC-00509 or KRC-00715 (Fig. 2). It indicates that our c-Met inhibitors don’t have any cytotoxic effect on c-Met low-expressed gastric cancer cells at all, but selectively suppress the growth of c-Met over-expressed gastric cancer cells, which is consistent with the previous reports [13]. However, unlike our c-Met inhibitors, crizotinib, a multi-kinase inhibitor, has shown cytotoxic effect on c-Met low-expressed cell lines to some extent. This difference is likely because of the selectivity to c-Met of compounds. This data strongly suggest that our c-Met inhibitors are more relevant for the targeted therapy for the c-Met over-expressed gastric cancer patients than crizotinib.

### c-Met inhibitors suppress c-Met auto-phosphorylation and its downstream signals in c-Met over-expressed gastric cancer cells

**Fig. 3:**
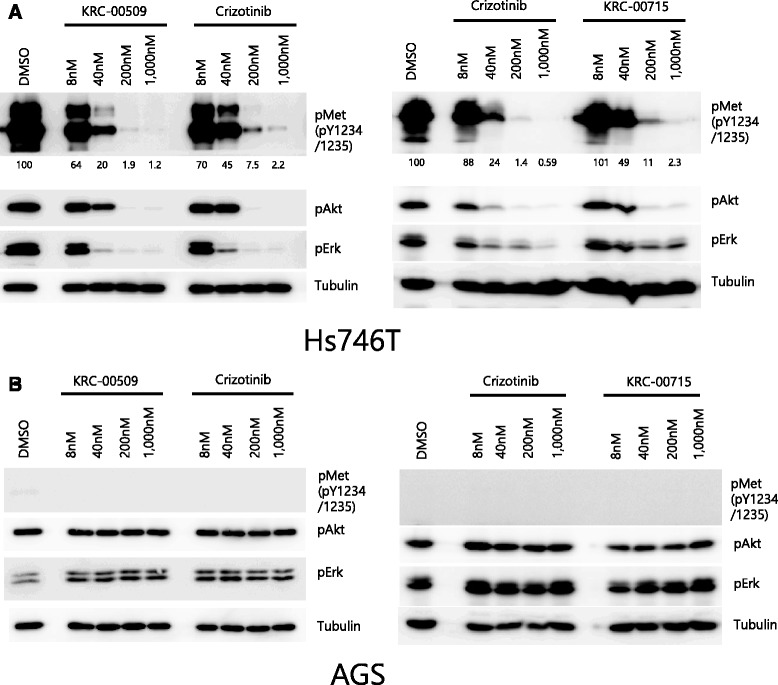
Phosphorylations of Akt and Erk are downregulated by c-Met inhibitors only in c-Met overexpressed cell lines. Hs746T (**a**), or AGS (**b**) was treated with c-Met inhibitors or crizotinib in a dose dependent manner for 3 h. Cell lysates were prepared for immunoblot with phospho antibodies of c-Met, Akt, and Erk. Tubulin band shows equal loading. c-Met phosphorylation was quantified by imageJ software

To investigate the cellular activity of inhibitors against human c-Met kinase, the level of phospho form of c-Met at tyrosine 1234/1235, which are auto-phosphorylation sites, was measured in Hs746T cell line. Cells were treated with each compound for 3 h, and lysates were prepared for western blot. As shown in Fig. 3a, c-Met auto-phosphorylations were greatly inhibited by c-Met inhibitors. KRC-00509 showed the most excellent inhibition against c-Met auto-phosphorylation. 8 nM KRC-00509 inhibited the c-Met auto-phosphorylation by 70 %. Crizotinib and KRC-00715 have less activity against c-Met auto-phosphorylation than KRC-00509. These data are consistent with the cell cytotoxic data (Table 1). This means that the inhibition of c-Met activity contributes mainly to the suppression of the Hs746T cell proliferation. To address the mechanism by which c-Met inhibitors block the cell proliferation, we checked the cellular phosphorylation of Erk and Akt (Fig. 3). In cancer cells addicted to c-Met signaling, the inhibition of c-Met activity results in the suppression of downstream signal pathways, such as Akt and Erk, which are important for cell survival and proliferation [6, 7]. Our data also showed that the phosphorylations of Akt and Erk were dramatically reduced by our c-Met inhibitors in c-Met over-expressed gastric cancer cells such as Hs746T, SNU-638, and SNU-620 (Fig. 3a, Additional file 1: Figure S2A-B). However, in c-Met low-expressed cell lines, such as AGS, SNU-1, and MKN-1, the phosphorylations of downstream signals were not influenced by c-Met inhibitors (Fig. 3b, Additional file 1: Figure S2C-D). These data mean our c-Met inhibitors are effective only to c-Met-addicted cells. Interestingly, Additional file 1: Figure S3 also shows that total cellular tyrosine phosphorylations in Hs746T were diminished by c-Met inhibitors, whereas in c-Met low expressed cell line, such as AGS, total cellular tyrosine phosphorylations were not affected. It means most tyrosine phosphorylations in c-Met- addicted cells are dependent on c-Met activity, which implicates the importance of c-Met in c-Met overexpressed cancer cells. Conclusively, inhibition of c-Met activity causes the suppression both of the important downstream signals and of the total cellular tyrosine phosphorylations in c-Met overexpressed cells leading to the cell death.

### c-Met inhibitors induce G1/S arrest to suppress the cell proliferation

**Fig. 4:**
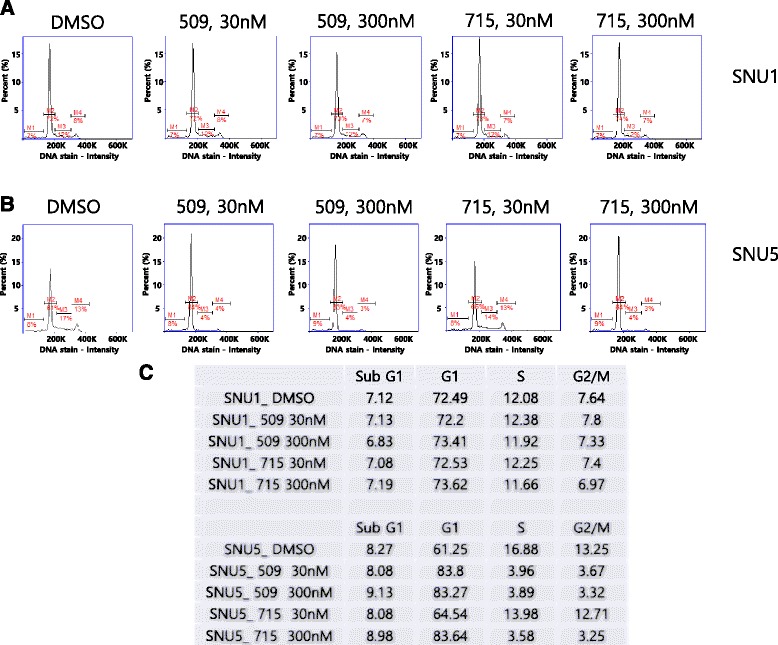
c-Met inhibitors induce G1/S arrest to suppress the proliferation of c-Met over-expressed cells. **a**-**b** SNU1 (**a**) or SNU5 (**b**) was treated with DMSO or c-Met inhibitors for 24 h. Cells were collected to be analyzed for cell cycle distribution by NucleoCounter NC-250 instrument according to the manufacture’s instruction. M1, M2, M3, and M4 indicate subG1, G1, S, G2/M phase respectively. **c** The populations of each phase are shown

To study how our c-Met inhibitors suppress the proliferation of c-Met over-expressed cells, we investigated the cell cycle distribution (Fig. 4). SNU1 and SNU5 were treated with our c-Met inhibitors for 24 h. SNU1 has low expression of c-Met, whereas SNU5 has high expression of c-Met as shown in Fig. 2. Cell cycle analysis shows cells treated with c-Met inhibitors were arrested at G1/S phase in SNU5. However, no cell cycle arrest was identified in SNU1. This data implies c-Met inhibitors suppress the cell proliferation by inducing G1/S arrest in c-Met over-expressed cells.

### KRC-00715 suppresses the tumor size in Hs746T mouse xenograft assay

**Fig. 5:**
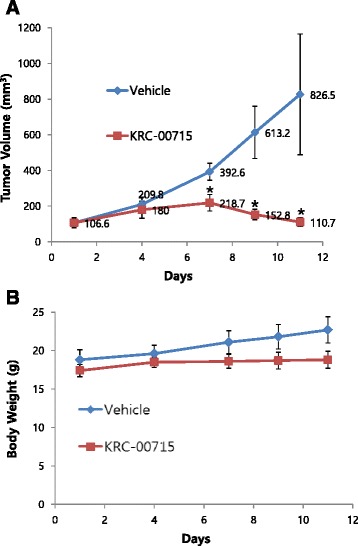
In vivo Hs746T xenograft assay. Hs746T cells were implanted into the mouse and allowed to grow to the designated size. Vehicle and KRC-00715 were orally administered to mouse daily at dose of 50 mpk. **a** Tumor sizes were measured using calipers throughout the treatment period. *, *P* ≤ 0.001 , median tumor volumes are significantly less in the treated versus the control group as determined using one-way ANOVA. **b** Weights were measured throughout the treatment period

To see if our c-Met inhibitors block the tumor growth in vivo, we used mouse Hs746T xenograft model. After Hs746T cells were implanted on nude mouse, we administered KRC-00509 and KRC-00715 orally at doses of 50 mpk daily when tumor size reaches to certain point (100 mm^3^ or 200 mm^3^). Tumor volumes were measured about for 10 days. KRC-00715 showed very dramatic result in vivo as shown in Fig. 5a. Tumor volumes were significantly reduced by KRC-00715. In addition, KRC-00715 administration to mouse didn’t cause any loss of weight (Fig. 5b). However, in vivo experiment with KRC-00509 was suspended as mice died 3–4 days after KRC-00509 administration. Conclusively, KRC-00715 is proved to have potent activity against c-Met in vivo as well as in vitro.

## Discussion

In this present study, we demonstrated a potent inhibitor of c-Met and its excellent cytotoxic effect on c-Met over-expressed gastric cancer cells. 10–20 % of gastric cancer tissues and 40 % of the scirrhous histological subtype have been known to harbor amplified *c*-*met* gene [14, 33, 34]. Our western blot also shows that 27 % (5/18) of gastric cancer cell lines have c-Met overexpression (Fig. 2a). Because cells with c-Met overexpression are known to be addicted to c-Met signaling, the strategy targeting c-Met may be a promising therapeutics for many of gastric cancer patients. Previous studies demonstrated c-Met inhibition, by siRNA or small molecule inhibitor, blocks the proliferation of *c*-*met* amplified cancer cell [12, 13, 35, 36]. We synthesized hundreds of compounds and performed in vitro biochemical assay. Our compounds have pyridoxazine instead of quinoline in PF-04217903 [30]. X-ray crystallography indicates that nitrogen of quinoline in PF-04217903 plays a role in hinge binding in a mono-binding manner. Based upon our molecular docking study, we synthesized triazolopyrazine compounds having pyridoxazine as a dual-binder to hinge region. By introducing pyridoxazine, we escaped the patent conflict with PF-04217903. KRC-00509 and KRC-00715 showed best efficacy in enzyme assay among the synthesized ones (Table 1, Additional file 1: Figure S1). Kinase panel assay demonstrated KRC-00715 is exclusively selective to c-Met like PF-04217903 (Table 2). It didn’t inhibit any tyrosine kinase at all at 1 μM. Therefore we expect our compound may reduce side effects to a minimal level in clinical trial. Our cytotoxic data supports this expectation by showing that cell lines with low level of c-Met weren’t suppressed by our compounds at all even at as high as 5 μM (Fig. 2). However, crizotinib, which is a multi-kinase inhibitor, cause cytotoxic effect on several c-Met negative cell lines to some extent. It implicates that crizotinib may cause side effects in clinical trials, and actually several clinical reports demonstrated it cause serious side effects including dermatitis, visual disturbance, heart problem, and so on [37, 38]. Table 1 indicates the cytotoxic effects of KRC-00509 and KRC-00715 on Hs746T were as good as crizotinib. Namely, our compounds, which inhibit c-Met exclusively, have the same cytotoxicity on c-Met-addicted cells as crizotinib which inhibits multi-kinases including c-Met. That’s why we think our compound may be a superior therapeutic agent to crizotinib for c-Met targeted therapy. Figure 3 shows why c-Met inhibitors had effects only on c-Met over-expressed cells not on c-Met low-expressed cells. Downstream signals, such as Akt, and Erk, were diminished by c-Met inhibitors only in c-Met over-expressed cells (Figure 3, Additional file 1: Figure S2). In addition, total cellular tyrosine phosphorylations were diminished by c-Met inhibitors only in c-Met over-expressed cells. (Additional file 1: Figure S3) These phenomena can be explained by ‘oncogene-addiction’. That is, c-Met over-expressed gastric cancer cells are ‘addicted’ to c-Met signaling. Therefore, c-Met-targeted therapeutics is quite relevant to gastric cancer patients with c-Met overexpression. One thing interesting is that c-Met inhibitors induced G1/S arrest in c-Met-addicted cells (Fig. 4) All of the successful agents for cancer targeted therapy, such as imatinib, sorafenib and gefitinib, have shown strong G1/S arrest to result in apoptosis [39–41]. If we get to know the exact mechanism how these agents cause G1/S arrest, it may widen our perception of cancer therapy. To see the in vivo efficacy of our compounds, we used mouse Hs746T xenograft model. KRC-00715 shows the significant tumor growth inhibition at doses of 50 mpk without loss of weight. However, we suspended the in vivo experiment with KRC-00509 because of the compound’s severe toxicity to mouse.

## Conclusions

In summary, we demonstrate newly synthesized c-Met inhibitor which is orally available. KRC-00715 is highly selective to c-Met, and shows excellent efficacy in vitro and in vivo.

## Additional file

Additional file 1: Figure S1. Inhibition Curve. The inhibition percentage was measured in c-Met enzyme assay. The detailed assay procedures are described in Methods. **Figure S2.** Phosphorylation of Akt and Erk is downregulated by c-Met inhibitors only in c-Met overexpressed cells. SNU-638 (A), SNU-620 (B), SNU-1 (C), or MKN-1 (D) cells were treated with KRC-00509 or crizotinib in dose dependent manner for 3 hr. Cell lysates were prepared for immunoblot with phospho antibodies of c-Met, Akt, and Erk. Tubulin band shows equal loading. **Figure S3.** Total tyrosine phosphorylations were reduced by c-Met inhibitors in c-Met overexpressed cells. Hs746T (A), or AGS (B) were treated with c-Met inhibitors or crizotinib in dose dependent manner for 3 hr. Cell lysates were prepared for immunoblot with phospho tyrosine antibody. (PPTX 6747 kb)

## Abbreviations

EGFR: Epidermal growth factor receptor
HGF: Hepatocyte growth factor
HTRF: Homogeneous time resolved fluorescence
PEG: Polyethylene glycol
PI3K: Phosphoinositide 3-kinase
RTK: Receptor tyrosine kinase
SA-XL665: Streptavidine-XL665
VEGF: Vascular Endothelial Growth Factor
