# Supplementary material for: Novel c-Met inhibitor suppresses the growth of c-Met-addicted gastric cancer cells
Source: BMC Cancer. 2016 Jan 22;16:35. doi: 10.1186/s12885-016-2058-y (PMC4722623; doi:10.1186/s12885-016-2058-y)
Supplement: Additional file 1: Figure S1. — Inhibition Curve. The inhibition percentage was measured in c-Met enzyme assay. The detailed assay procedures are described in Methods. Figure S2. Phosphorylation of Akt and Erk is downregulated by c-Met inhibitors only in c-Met overexpressed cells. SNU-638 (A), SNU-620 (B), SNU-1 (C), or MKN-1 (D) cells were treated with KRC-00509 or crizotinib in dose dependent manner for 3 hr. Cell lysates were prepared for immunoblot with phospho antibodies of c-Met, Akt, and Erk. Tubulin band shows equal loading. Figure S3. Total tyrosine phosphorylations were reduced by c-Met inhibitors in c-Met overexpressed cells. Hs746T (A), or AGS (B) were treated with c-Met inhibitors or crizotinib in dose dependent manner for 3 hr. Cell lysates were prepared for immunoblot with phospho tyrosine antibody. (PPTX 6747 kb) [file 12885_2016_2058_MOESM1_ESM.pptx]

## Slide 1
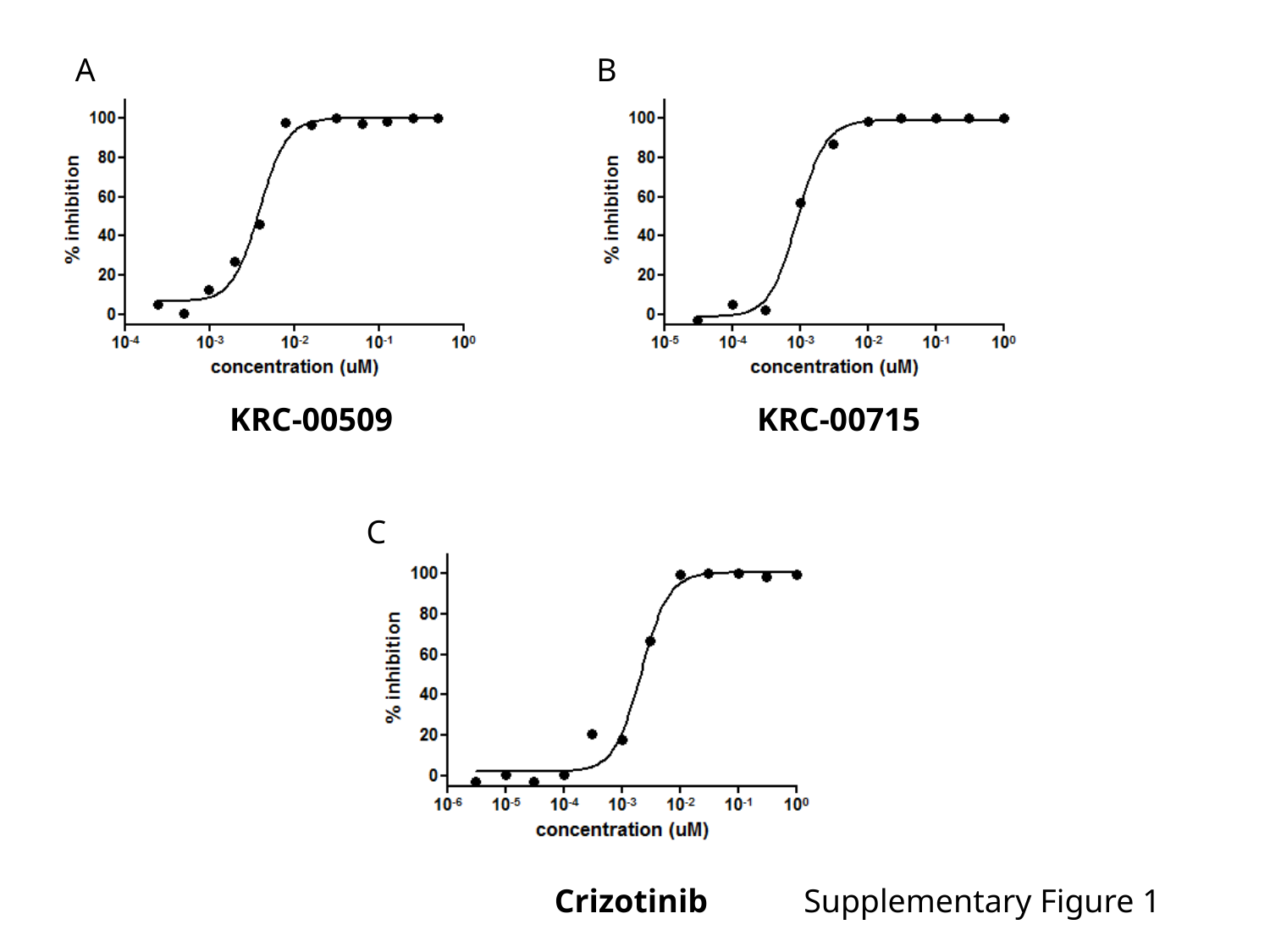

A
B
KRC-00509
KRC-00715
C
Crizotinib
Supplementary Figure 1

## Slide 2
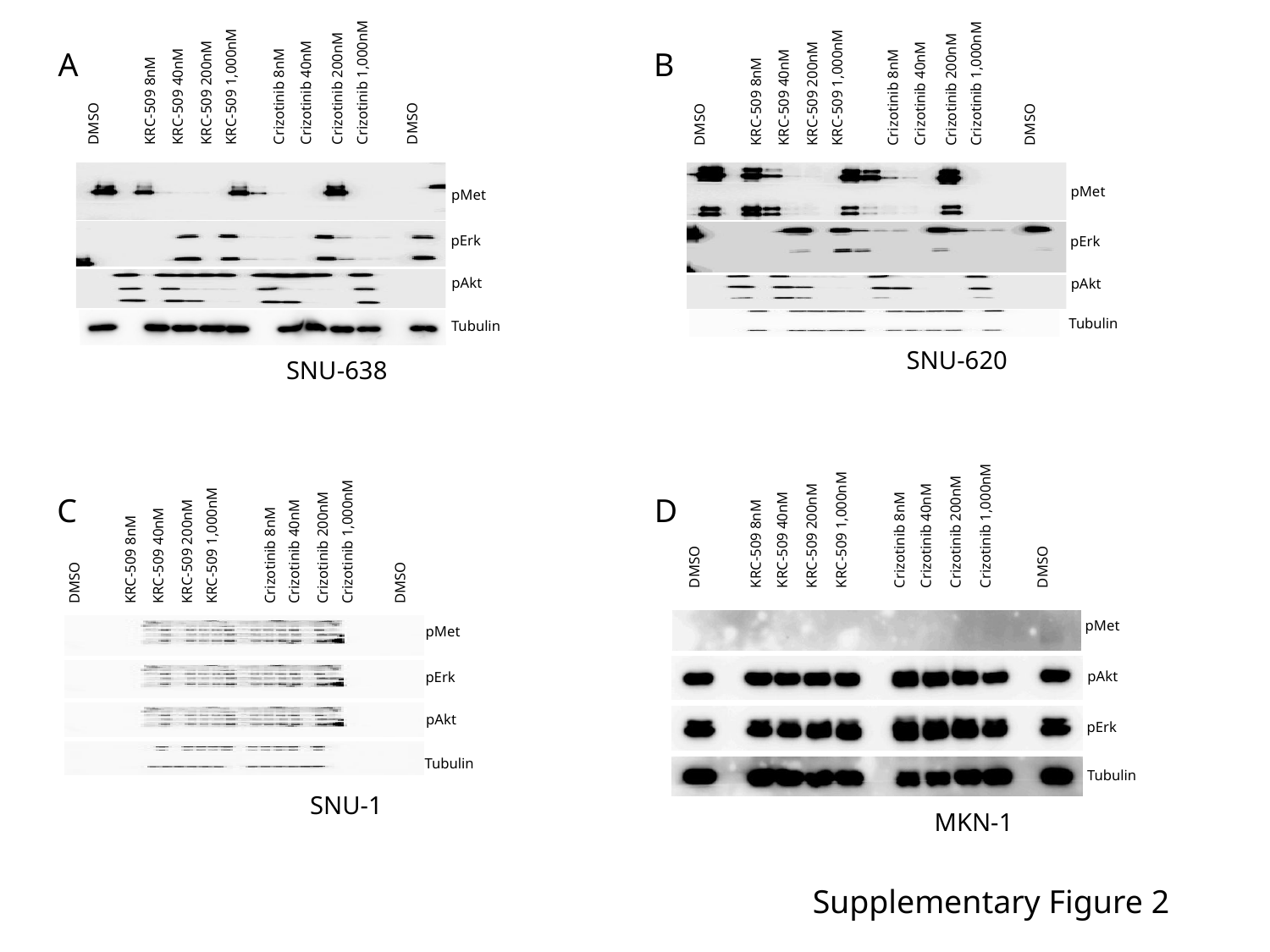

A
Crizotinib 1,000nM
B
Crizotinib 1,000nM
KRC-509 1,000nM
KRC-509 1,000nM
Crizotinib 200nM
Crizotinib 200nM
KRC-509 200nM
KRC-509 200nM
Crizotinib 40nM
Crizotinib 40nM
KRC-509 40nM
KRC-509 40nM
Crizotinib 8nM
Crizotinib 8nM
KRC-509 8nM
KRC-509 8nM
DMSO
DMSO
DMSO
DMSO
pMet
pMet
pErk
pErk
pAkt
pAkt
Tubulin
Tubulin
SNU-620
SNU-638
Crizotinib 1,000nM
KRC-509 1,000nM
Crizotinib 200nM
KRC-509 200nM
Crizotinib 40nM
C
D
KRC-509 40nM
Crizotinib 8nM
Crizotinib 1,000nM
KRC-509 8nM
KRC-509 1,000nM
Crizotinib 200nM
KRC-509 200nM
Crizotinib 40nM
KRC-509 40nM
Crizotinib 8nM
KRC-509 8nM
DMSO
DMSO
DMSO
DMSO
pMet
pMet
pAkt
pErk
pAkt
pErk
Tubulin
Tubulin
SNU-1
MKN-1
Supplementary Figure 2

## Slide 3
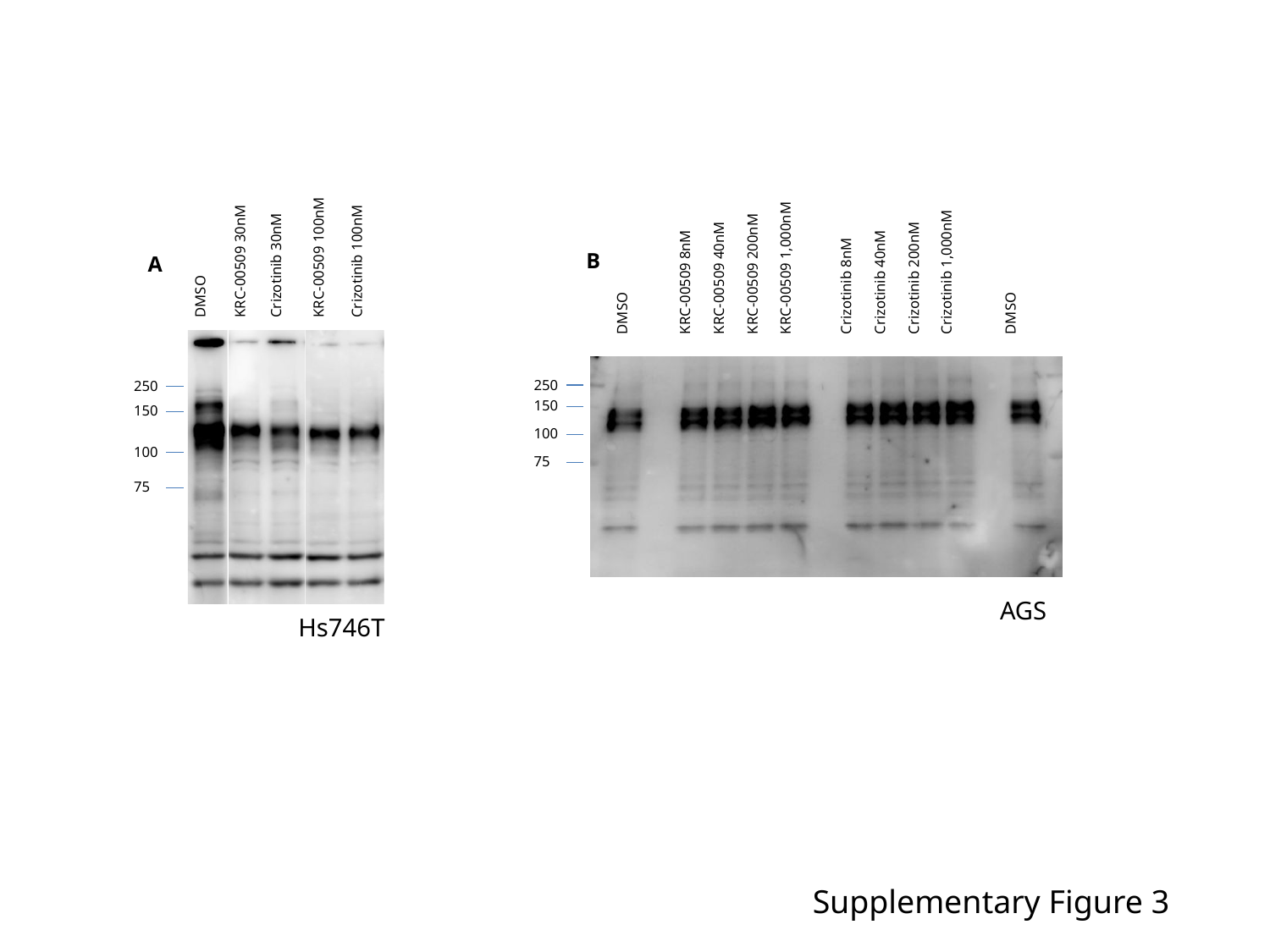

KRC-00509 100nM
KRC-00509 1,000nM
KRC-00509 30nM
Crizotinib 100nM
Crizotinib 1,000nM
KRC-00509 200nM
Crizotinib 30nM
KRC-00509 40nM
Crizotinib 200nM
KRC-00509 8nM
Crizotinib 40nM
Crizotinib 8nM
B
A
DMSO
DMSO
DMSO
250
250
150
150
100
100
75
75
AGS
Hs746T
Supplementary Figure 3
